# Learning to perceive and recognize a second language: the L2LP model revised

**DOI:** 10.3389/fpsyg.2015.01000

**Published:** 2015-08-04

**Authors:** Jan-Willem van Leussen, Paola Escudero

**Affiliations:** ^1^Amsterdam Centre for Language and Communication, University of AmsterdamAmsterdam, Netherlands; ^2^The MARCS Institute and ARC Centre of Excellence for the Dynamics of Language, University of Western SydneySydney, NSW, Australia

**Keywords:** non-native perception, non-native recognition, second-language learning, computational modeling

## Abstract

We present a test of a revised version of the Second Language Linguistic Perception (L2LP) model, a computational model of the acquisition of second language (L2) speech perception and recognition. The model draws on phonetic, phonological, and psycholinguistic constructs to explain a number of L2 learning scenarios. However, a recent computational implementation failed to validate a theoretical proposal for a learning scenario where the L2 has less phonemic categories than the native language (L1) along a given acoustic continuum. According to the L2LP, learners faced with this learning scenario must not only shift their old L1 phoneme boundaries but also reduce the number of categories employed in perception. Our proposed revision to L2LP successfully accounts for this updating in the number of perceptual categories as a process driven by the meaning of lexical items, rather than by the learners' awareness of the number and type of phonemes that are relevant in their new language, as the previous version of L2LP assumed. Results of our simulations show that *meaning-driven* learning correctly predicts the developmental path of L2 phoneme perception seen in empirical studies. Additionally, and to contribute to a long-standing debate in psycholinguistics, we test two versions of the model, with the stages of phonemic perception and lexical recognition being either sequential or interactive. Both versions succeed in learning to recognize minimal pairs in the new L2, but make diverging predictions on learners' resulting phonological representations. In sum, the proposed revision to the L2LP model contributes to our understanding of L2 acquisition, with implications for speech processing in general.

## Introduction

Adult second language (L2) learners often struggle to understand native speech and to make themselves understood by native speakers. One important reason behind this difficulty seems to be that adult learners rely on the rules and categories of their own native language (L1) when learning to perceive and produce L2 sounds. Numerous experiments have demonstrated the influence of L1 perception and the specific problems it causes for L2 learners: for instance, troublesome English minimal pairs are “rocket” and “locket” for Japanese speakers (Aoyama et al., 2004), “beat” and “bit” for Spanish (Flege et al., 1997) and Portuguese (Rauber et al., 2005) speakers, or “bet” and “bat” for Dutch speakers (Broersma, 2005). The overarching cause of these problems is that these specific sounds do not contrast in these learners' L1 phoneme repertoires. In other words, novel L2 contrasts are difficult to perceive and produce.

Linguistic experience is therefore at the core of current theories and models of L2 perception and production, which advance proposals and predictions based on how L1 speech sounds compare to those in the new language. Three such theories, the Perceptual Assimilation Model (PAM; Best, 1995) and its extension to L2 learning (PAM-L2; Best and Tyler, 2007), the Speech Learning model (SLM; Flege, 1995; Flege et al., 2003) and the Second Language Linguistic Perception model (L2LP; Escudero, 2005, 2009) explain how L1 experience influences L2 sound learning in a number of learning scenarios. We sketch three such scenarios and their predicted result in L2LP, SLM, and PAM-L2 below. Unlike the other two models which account for either naïve non-native and beginning L2 perception (PAM and PAM-L2) or L2 speech learning (SLM), as reviewed in Tyler et al. (2014), L2LP aims at modeling the entire developmental process of L2 speech perception, from naïve, non-native to advanced, native-like performance. L2LP therefore proposes precise learning tasks and developmental trajectories for learners, depending on the learning scenario with which they are confronted, and comes with a computational learning model within the connectionism-inspired learning framework of Stochastic Optimality Theory (Boersma, 1998).

The basis for all predicted L2 learning trajectories in L2LP is the *optimal perception hypothesis* (Escudero, 2005, 2009). This states that learners will initially perceive L2 sounds in a manner resembling the production of these same sounds in their L1 environment. The L2LP model thus explicitly represents the *result* of L1 acquisition as the *initial state* of L2 learning, predicting that acoustical differences and similarities between the phonemes of two languages will shape development. From this starting point, three scenarios can be distinguished. Unlike the SLM which deals with isolated L2 sounds, both the L2LP and PAM make predictions for the perceptual development of sound contrasts. When the majority of productions of an L2 contrast are acoustically closest to typical or average productions of a single L1 sound, learners face what L2LP calls a new scenario and PAM calls *single category assimilation* (Best, 1995). Learners facing this scenario must either create a new L2 category or split their existing single L1 category. L2LP and PAM predict that this is a difficult scenario for L2 learners, and the experimental studies cited above confirm this. In contrast, when the majority of the tokens of an L2 contrast are acoustically closest to the typical productions of two separate L1 sounds, learners are faced with a similar scenario (PAM: two-category assimilation). According to the L2LP, in this scenario, the existing L1 categories are simply replicated and then adjusted so that their boundaries will come to match those of the L2 contrast, as there is hardly ever a perfect match between the productions of an L1 and L2 contrast. PAM and L2LP predict that this shifting is less problematic than creating new categories (Escudero et al., 2014), while Flege's SLM predicts that new sounds would be easier to learn than similar or old sounds (Flege, 1995). However, since the SLM focuses on single sounds and not on sound contrasts, as the PAM and L2LP models, a comparison of predictions across models may not be straightforward.

A third possible case only considered by the L2LP and PAM is the subset scenario, which may be comparable to what is called *uncategorized* or *categorized-uncategorized assimilation*, depending on how each of the members of the contrast are *assimilated* to native categories, in PAM. It takes place when a single non-native sound is perceived as more than one L1 category, so-called *multiple category assimilation* within the L2LP (Escudero and Boersma, 2002; Escudero, 2005). Both the PAM and L2LP models predict that this scenario poses fewer problems than the new scenario, since no new contrast has to be created in L2 perception (L2LP) and little discrimination difficulty is predicted (PAM). Given that the PAM and PAM–L2 use perceptual assimilation data to make predictions for discrimination accuracy, while they do not predict assimilation patterns (Escudero et al., 2014; Tyler et al., 2014; Colantoni et al., 2015, Chapter 2), these models would predict little discrimination difficulty for Dutch learners of Spanish from Escudero and Boersma's (2002) categorization pattern. This is because as reported in Escudero and Boersma (2002, Table 1), Dutch listeners perceived Spanish /i/ mostly as Dutch /i/ (in average 71% of 25 tokens) and Spanish /e/ mostly as Dutch /i/ (in average 65% of 25 tokens), which according to PAM would lead to a two-category assimilation or a category-goodness scenario^1^, resulting in very good to good discrimination. However, L2LP's architecture allows pinpointing a potential difficulty for learners in this scenario that goes beyond discrimination difficulty: Escudero and Boersma (2002) note that if a learner's L1 contrasts are left intact when acquiring an L2 without this contrast, this may in turn lead to spurious contrasts at the word level (i.e., lexical contrasts), ultimately hampering the attainment of a fully native-like command of the L2.

If the purpose of speech communication is to understand and to be understood, it seems important to not only concentrate on how perceptual development takes place in an L2 but also to examine how the novel L2 categories are employed to recognize and store new words in the L2 lexicon. Experimental evidence suggests continuity between L2 perceptual and lexical abilities, as difficulties in distinguishing novel L2 sounds are commonly accompanied by difficulties in distinguishing L2 minimal pairs. However, other research has shown dissociation between perceptual and lexical abilities in L2 development. For instance, some studies document that L2 learners fail to encode a novel L2 contrast lexically, despite them being fully able to perceive the L2 contrast (e.g., Curtin et al., 1998), while other studies show that the opposite can also be true: L2 learners may develop distinct lexical representations for words that they cannot reliably discriminate in perception (Weber and Cutler, 2004; Cutler et al., 2006; Escudero et al., 2008) or production (Hayes-Harb and Masuda, 2008). These studies suggest that distinguishing pre-lexical perception from lexical recognition in an L2 model will provide further insight into the processes underlying L2 acquisition. By incorporating separate but linked representations for *perceptual* and *lexical* contrast, L2LP can serve as a model to investigate both continuity and discrepancy between perceptual and lexical abilities in L2 acquisition.

The computational architecture of L2LP allows simulating the entire trajectory from naïve to experienced L2 listener in various scenarios. These trajectories can then be compared to empirical data to assess the adequacy of the model. Escudero and Boersma (2004) and Escudero (2009) performed simulations with computer-modeled learners in the L2LP framework, showing that these exhibited developmental paths that are comparable to the performance of Spanish learners of the Southern British (SBE) and Scottish English (SE) /i/−/i/ contrast. These learners face a new and similar scenario respectively, as exemplars of the vowels in SBE are acoustically closest to Spanish /i/, while exemplars of SE are acoustically closest to /i/ and /e/. However, the modeled learners in these studies had direct access to the phonemic or phonological categories of the L2 in the input data. Escudero (2005) argues that ultimately L2 learning should be modeled as *meaning-driven* or *message-driven*^2^: learners have no direct access to the phonological categories employed by native speakers of the L2, but rather infer these based on how well they are able to understand the meaning intended by a speaker. This is, in fact, a more ecologically valid proposal. Escudero's theoretical account of this more realistic mechanism for language learning used the subset scenario for Dutch learners of the Spanish /i/-/e/ contrast as a case study. Dutch has three front vowels /i/, /i/, and /ε/ in the area of the vowel space where Spanish has only /i/ and /e/, which according to the L2LP should lead to the multi-category assimilation of Spanish /i/ as Dutch /i/ and /i/ and Spanish /e/ as Dutch /i/ and /ε/, which was confirmed in in naïve, beginning, intermediate, and advanced Dutch learners of Spanish (Escudero and Boersma, 2002). The theoretical account predicted that meaning-driven learning would result in a reduction of the middle /i/ category. However, a computational implementation of the model by Weiand (2007) failed to confirm this hypothesis, as the modeled learners mostly did not manage to converge on a more L2-like grammar. A thorough inspection of Weiand's results has led us to believe that by revising some details of learning and representation in the model, meaning-driven category *reduction* could be borne out.

In the present study, we further investigate the adequacy of the L2LP model, in its theoretical proposal (Escudero, 2005) and earlier computational implementations (Escudero and Boersma, 2004; Weiand, 2007; Boersma and Escudero, 2008), for explaining a case of perception and lexicalization of an L2 contrast. Although PAM-L2 incorporates the role of the lexicon in L2 sound perception, it is limited to hypothesizing that vocabulary size determines L2 sound perception success. Current psycholinguistic models of spoken-word recognition (e.g., McClelland and Elman, 1986; Norris, 1994; Gaskell and Marslen-Wilson, 1997) assume that the process of identifying a word in the lexicon is the result of a process of competition between lexical candidates that are activated at the same time, with each candidate being supported to different degrees by the speech signal. L2LP uses this activation process in a network-like model. Another important feature of L2LP that is compatible with a number of L1 acquisition models (e.g., PRIMIR, Werker and Curtin, 2005) is the assumption of continuity between perceptual and lexical development: perceptual learning is triggered as learners attempt to improve recognition by updating their lexical representations. This trickle-down view of *meaning-driven lexical learning* and *lexicon-driven perceptual learning* will be detailed below. In short, L2LP bridges insights from the field of L2 sound acquisition with more general cognitive theories of linguistic processing. These concepts are embedded in a simulation framework that is capable of generating quite specific predictions for various acquisition scenarios.

The present study has two aims. First, we present a revised version of L2LP, changing two crucial details of how learning takes place but retaining the fundamental properties of the model listed above. We assess the explanatory adequacy of this revised L2LP by re-applying it to an instance of lexical and perceptual learning in the Dutch-to-Spanish subset scenario described above. Our hypothesis is that the revisions will improve the L2LP's ability to model the learning process in a multiple-category assimilation case followed by a subset scenario, as observed in real L2 learners by Escudero and Boersma (2002).

Second, we propose two alternative versions of the revised model with regards to information flow from speech signal to lexicon, given that pre-lexical and lexical perception can be implemented as *sequential* (strictly bottom-up) or *interactive* (allowing lexical feedback to lower-level perception). The existence of lexical feedback is a matter of much debate within models of psycholinguistics, as shown by Norris et al.'s (2000) proposal and the many alternatives that emerged in response McClelland et al., 2006; McQueen et al., 2006. In Escudero (2005)'s account, L2 comprehension is described as sequential, but this is not a necessary property of the model. We will thus contribute to this more general debate by investigating the explanatory adequacy of these alternative views on processing grammars in L2 speech comprehension. Below, we present our revised version of the L2LP model and its specific application to the subset scenario, and demonstrate that it successfully explains L2 learning of perception and lexicalization.

## The L2LP model revised

Escudero's (2005) L2LP model aims at providing a comprehensive platform to explain L2 acquisition, perception, and lexicalization. It grew out of, and co-evolved with, the Bidirectional Phonetics and Phonology framework (Boersma, 1998, 2011; henceforth BiPhon), which itself is an extension of Optimality Theory (OT; Prince and Smolensky, 1993). In this section, we describe how linguistic knowledge, processing, and learning are implemented in a revised version of L2LP, taking care to highlight changes from Escudero (2005)'s description and Weiand (2007)'s implementation.

### Architecture of the L2LP-revised: levels and connections

**Figure 1:**
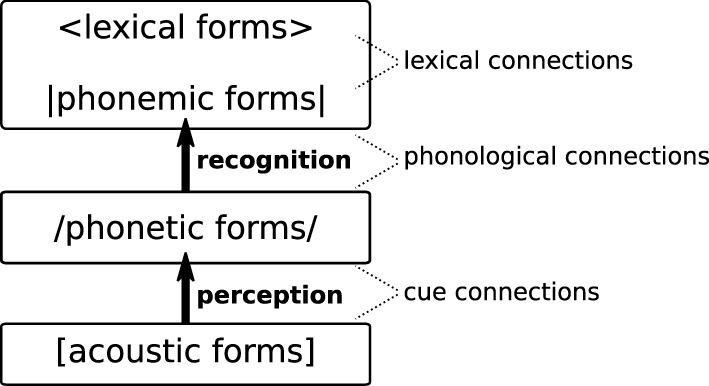
**The levels of representation and connection types in the L2LP model**.

Like its predecessors, L2LP is an explicit *computational* model of the processes driving L2 perception and learning. Modeling the acquisition of pre-lexical phonetic categorization in the L2, as well as the subsequent recognition of L2 categories in stored lexical items, requires units on four levels of representation. Figure 1 shows an overview of these four levels and the connections between them.

At the bottom we find the *acoustic* level, representing incoming speech sounds as they arrive in the peripheral auditory system. The subsequent *phonetic* level encodes a speaker's language-specific, invariant representations of speech sounds, including context-specific allophonic detail. These intermediate representations are linked to the *phonemic* level where possible canonical forms of words/morphemes are stored, encoding only contrasts that may change the meaning of a word. Finally, phonemic forms connect to possible meanings at the *lexical* level ^3^. By including an intermediate phonetic level between the acoustic signal and phonemic forms stored in the lexicon, L2LP aims to explicitly represent the distinction between the pre-lexical and lexical stages of speech perception, as was described in the Introduction.

Units on adjacent levels are connected, and the process of perceiving and eventually recognizing an incoming word is represented in the model as a four-step path through this network: [acoustic] → /phonetic/ → |phonemic| → <lexical>. The winning or *optimal* path is decided by relative strength of connections among competing paths. While the units themselves are fixed, the strengths of the connections are altered over the course of learning. This in turn alters the optimal paths from acoustics to lexicon through the network. Knowledge of a language is thus stored in the connection strengths: for instance, a strong |phonemic| → <lexical> connection encodes knowledge of a given lexical item as a meaning-form pair.

A central assumption of L2LP is the *Full Copying* hypothesis (Escudero, 2005): L2 learners initiate their learning process on a duplicate or copy of their L1 perception grammar, so that their L2 grammar is attuned to the sounds and categories of the L1. Over time, exposure to the new language shifts the connections of this copy to a state more suited to perception and recognition of the L2. The next sections elaborate this learning process, showing how perception, recognition, and learning are modeled in the Dutch to Spanish subset scenario that is the focus of this paper.

### Evaluating optimal paths

**Figure 2:**
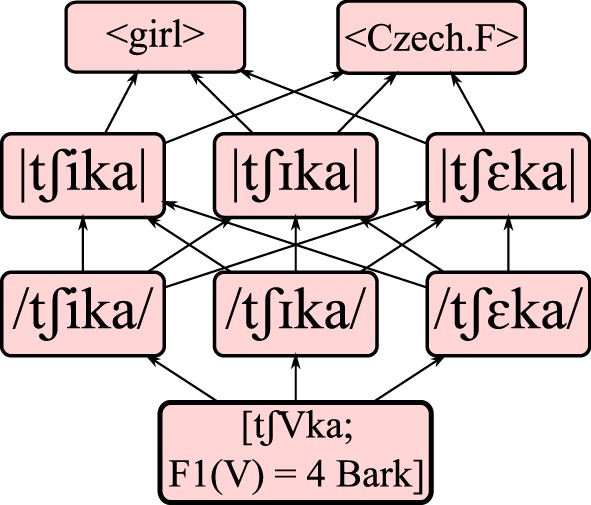
**Possible mappings for the input [tʃVka; F1(V) = 4 Bark], via phonetic and phonemic representations, to a lexical form**. Each bottom-to-top path through the graph represents a possible pathway of perception and recognition.

An incoming word is represented as a unit on the [acoustic] level. As this study concerns the L2 acquisition of Spanish *front vowels*, inputs are represented by two variables, namely a “carrier” word containing a front vowel, and the first formant (F1) of the said vowel, which is the acoustic cue for vowel height. The carrier words (see Appendix) are always members of a Spanish /i/-/e/ minimal pair, and are represented as acoustically invariant: they can be seen as narrowing down the available units in the network to those specific to a given minimal pair. The F1 input values do show acoustic variation: they are represented as discretized values on the psychoacoustic Bark scale, ranging from 2 to 8 Bark in steps of 0.1 Bark. For example, the acoustic input [tʃVka, F1(V) = 4.0 Bark] corresponds to a realization of either the Spanish word *chica* “girl” or of *checa* “Czech female,” with an F1 value of 4 Bark for the front vowel (V). Figure 2 shows the possible mappings from this particular input form, via phonetic and phonemic representations, to one of two possible lexical meanings. All other combinations of carrier words and front vowel realizations are similarly connected to two possible meanings via the two intermediate levels of representation.

Under the assumption that the L2 grammar is initially a copy of the L1 grammar, the learner may connect the [V] contained in the acoustic input to one of the three different front vowels of Dutch on the /phonetic/ level, embedded in a phonetic representation of the carrier word. Our example input [tʃVka, F1(V) = 4.0 Bark] thus connects to the phonetic representations /tʃika/, /tʃika/ and /tʃεka/. These connect in turn to three phonemic representations |tʃika|, |tʃika|, and |tʃεka|, which lead to either of two <lexical> items, namely <girl> or <Czech.F>. This yields a total of 18 paths (3 × 3 × 2) from acoustics to lexicon for each representable acoustic input. The relative strengths of connections along the paths decide the optimal route. However, the *ranking values* encoding these connection strengths are distorted slightly at each evaluation step by adding a random value from a normal distribution. This *stochastic* evaluation (Boersma, 1998) allows the model to deal with probability and variation when mapping from input to output. Stochastic evaluation is also robust to occasional errors in the input data during the learning procedure (detailed in Section Meaning-driven Learning below), making it more likely to converge on a target language (Boersma and Hayes, 2001).

**Figure 3:**
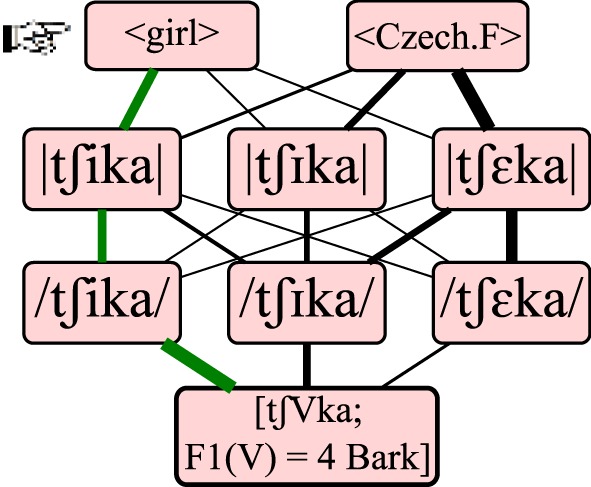
**Recognizing a lexical form by finding an optimal path**. Of the 18 possible routes from sound to meaning, the optimal path is that whose weakest connection is stronger than the weakest connection of any other path. In this figure, line thickness visualizes connection strength. The input containing a front vowel with an F1 of 4 Bark is perceived as phonetic /tʃika/, phonemic |tʃika|, and ultimately recognized as lexical <girl>.

Following a central tenet of Optimality Theory, the optimal path from [acoustic] to <lexical> is not defined by the *sum* of its connection strengths. Rather, a path is as strong as its weakest link, which means that the optimal path is the one containing the least weak connections. Equivalently, one can envision evaluation as iterating through the connections from weakest to strongest, pruning each connection until a single route remains. Figure 3 illustrates this evaluation procedure and is further explained below.

The [acoustic]-/phonetic/ connection strengths are initially inherited from the L1 Dutch grammar and thus suitable for the Dutch system with three front vowels. The /phonetic/ → |phonemic| connections are also not arbitrary, as the grammar is biased toward what phonologists refer to as *faithful* mappings, i.e., the connections between a phonetic representation and its “identical” phonological counterpart (e.g., /i/ → |i|, /i/ → |i| and /ε/ → |ε|). The bias is enforced by initializing these connections as stronger than the other six /phonetic/ → |phonemic| connections. Nevertheless, this is an important conceptual shift from the original architecture proposed by Escudero (2005) and its implementation by Weiand (2007). While they also biased the grammars toward faithful mappings, this bias was *qualitative*, so that these connections could never be weaker than a non-faithful mapping, and their strength was impervious to learning. In the revised L2LP, this initial bias is *quantitative* and may diminish or vanish over the course of learning. Thus, our revision retains symbolic representations but has a connectionist perspective on the relation between the /phonetic/ and |phonemic| levels: the two types of representation are of a distinct nature, there is no identity mapping, and the affinities between units on the two levels are gradual.

Finally, the |phonemic| → <lexical> connections are all initialized at equal strength in the L2 grammar, since no knowledge about the lexical meaning of Spanish word forms could be inherited from the L1 grammar. While |phonemic| → <lexical> mappings are specific to the subnetworks selected by the carrier words, the [acoustic] → /phonetic/ and /phonetic/ → |phonemic| connection strengths pertain only to the representations of front vowels and are shared between representations regardless of carrier word. An update triggered by our example acoustic input [tʃVka, F1(V) = 4.0 Bark] will therefore also affect the outcome of all other inputs with an F1 of 4 Bark; at the same time the updating of |phonemic| → <lexical> connection strengths affects the outcome for the carrier word [tʃVka] across all F1 input values. This update to both levels of connections triggered by an acoustic input validates the need for both a phonemic and a lexical level within the model.

### Sequential vs. interactive processing

As discussed in the Introduction, a standing debate in cognitive models of speech processing is whether the outcome of (pre-lexical) perception forms the input to recognition, or whether the two processes are performed in parallel and may interact with one another. Escudero (2005)'s theoretical treatment of L2LP and its implementation by Weiand (2007) is sequential: their learners always evaluate the [acoustic] → /phonetic/ connections of perception before the /phonetic/ → |phonemic| and |phonemic| → <lexical> connections of recognition. However, this two-step processing is not a necessary feature of the model, as Boersma (2011) shows that BiPhon (and by extension L2LP) can handle interaction between different levels of representation. By removing the strict ordering of connections in evaluation, recognition may interact with perception.

In our implementation, assigning connections *stratum* indices besides their ranking value enforces strict sequential ordering. At evaluation time, connections are ordered first by stratum, then by (distorted) ranking value. This means that if we place the [acoustic] → /phonetic/ connections in a higher stratum, perception precedes recognition and we simulate a learner with sequential perception and recognition. Conversely, by placing all connections in the same stratum, the connections of recognition may influence the outcome of perception. This allows us to compare a purely *bottom-up* version to an *interactive* version of the model, all else being equal.

### Meaning-driven learning

Learning in the L2LP framework equates with updating the connection strengths in the network, and is *error-driven*: simulated learners attempt to improve perception and recognition of the L2 whenever the current state of the grammar leads to misunderstandings. This is referred to as meaning-driven learning, as described above. After an acoustic input is evaluated and matched to a lexical form (Section Evaluating Optimal Paths), the learner is presented with a *target* <lexical> form encoding the intention of the speaker. If this target form matches the lexical form as understood by the learner, recognition is correct and no action is undertaken. In case of a mismatch, the learner will attempt to decrease the likelihood of a future mismatch by updating their grammar through weakening all connections along the path that led to the incorrect lexical form, and strengthening all connections along the path to the intended target form. If the two paths share subpaths, the net change in the strength of that connection will be zero. The *plasticity* value that is subtracted and added in order to weaken and strengthen connections, respectively, gradually decreases during learning.

Importantly, the target <lexical> item presented to learners contains no information on the /phonetic/ or |phonemic| categories employed by the speaker. The connection strengths on these intermediate levels must be updated such that future instances of this acoustic input will follow a path to the intended target item. Although the use of minimal pairs restricts possible outputs to two <lexical> items, the learner is confronted with several possibilities for performing this update, and is initially biased toward retaining its three-vowel L1 Dutch system where possible.

**Figure 4:**
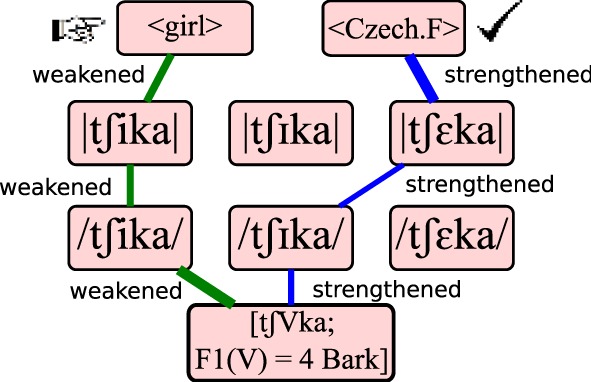
**Error-driven learning**. The learner discovers that it should have recognized lexical <Czech.F> rather than <girl>. It performs another evaluation, this time within the subset of paths leading to <Czech.F>. Learning strengthens connections along that path, and weakens connections along the incorrect path initially found.

Since nine distinct paths lead from any input to each individual lexical form, the learner must first *parse* a single path to the correct form to decide which connections to strengthen. Finding this parse occurs through *interpretive parsing* (Tesar and Smolensky, 1998). That is, the learner uses its current grammar to find an alternative path, but this time considers only the subset of nine paths leading to the target form, instead of the full network, as shown in Figure 4. Following Jarosz (2013), and departing from the implementation of Weiand (2007), evaluation noise is re-applied to the connections prior to parsing. Jarosz found that this “resampling” technique greatly increases the chances of finding a grammar that is compatible with the input data in Optimality-Theoretic, error-driven learning models.

To summarize, the present L2LP-revised model implements Escudero (2005)'s proposal with the following three revisions: (1) the phonologically inspired bias for “faithful” mappings is less restrictive (Section Evaluating Optimal Paths), (2) the possibility of interaction between perception and recognition can be explored (Section Sequential Vs. Interactive Processing), and (3) Jarosz (2013)'s resampling is applied in parsing to enhance the likelihood of convergence. The next section describes the methodology for training and testing our model of the subset scenario using computational simulations.

## Computational modeling with the L2LP-revised model

We performed a number of learning simulations to investigate whether the revised model described in Section The L2LP Model Revised can successfully implement the meaning-driven SUBSET learning scenario described by Escudero (2005). The simulation program consisted of two phases: *L1 training*, in order to create the “naïve” L1 starting point from which L2 acquisition proceeds, and *L2 training* to simulate the acquisition of Spanish categories through error-driven learning on lexical items. This two-stage simulation procedure was applied both for sequential-type learners whose [acoustic]-/phonetic/ connections are always evaluated before all other connections, and for interactive-type learners whose connections pertaining to recognition are allowed to outrank connections pertaining to perception. At various points during both training procedures, learners were given data from a test set in order to investigate to what extent L2 training improves recognition of the Spanish lexical items, as well as how phonemic/phonetic categories were remapped to this end.

Parameter settings were identical to those used in Boersma and Escudero (2008) and Weiand (2007) wherever possible. *Ranking values* (strength) for all connections were initialized to an equal value of 100, with the exception of /phonetic/-|phonemic| connections, which were set to 95 for “faithful” connections that preserved identity across these levels (Section Evaluating Optimal Paths), and to 105 for the other connections. The *evaluation noise* parameter was set to 2.0, which represented the standard deviation of a random normal distribution (centered around zero) that distorted ranking values before each evaluation. *Plasticity* was initialized to 0.1 at the start of learning, with a *decay rate* such that plasticity shrank by a factor 0.7 every 10,000 steps.

### Acoustic input data for the simulated learners

In both training phases, simulated learners were repeatedly given [acoustic] inputs, each of which represented some word or utterance containing a front vowel. The auditory correlate of the *height* of these front vowels is its first formant (F1), which the grammar represents on the psychoacoustic Bark scale, from 2.0 to 8.0 Bark in bins of 0.1 Bark. In order to increase the ecological validity of our simulations, we obtained these F1 values from two recent, methodologically similar vowel production studies, as described below.

The F1 values for the L1 Dutch input data were generated by taking all female tokens of the vowels /i/, /i/, and /ε/ from the corpus of van Leussen et al. (2011), converting the F1 of these tokens to Bark and rounding it to the nearest “bin.” The L2 formant values were likewise generated by taking all female tokens of /i/ and /e/ from Chládková et al. (2011), but these were also paired with a randomly selected carrier word containing either /i/ or /e/ in Spanish. Carrier words were the minimal pairs listed in the Appendix, which were the same as those used in Weiand (2007).

**Figure 5:**
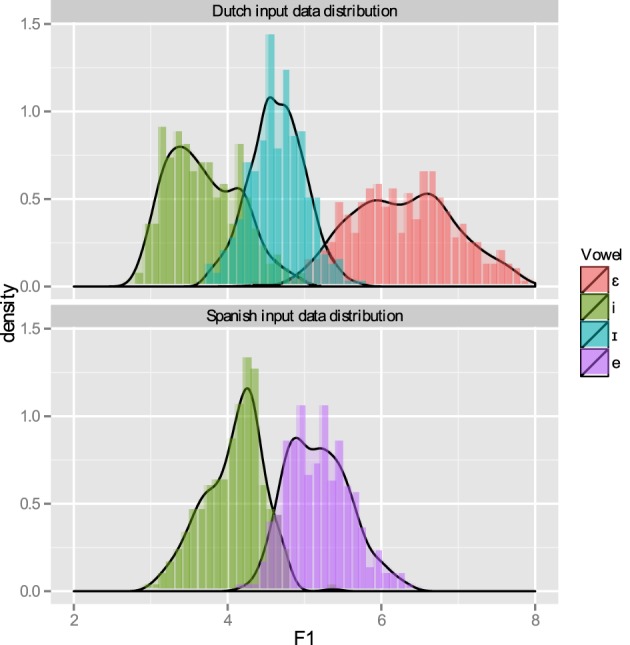
**Distribution of input data over the F1 continuum for Dutch (above) and Spanish training phases**. The histograms represent the “binned” input data.

Figure 5 shows the distribution of the F1 per category in the L1 and L2 input data.

### Training and testing procedures

In the L1 Dutch training phase, simulated learners were exposed to [acoustic] ~ /phonetic/ pairs of binned input F1 values and target vowels, in order to train them directly on the three-way Dutch contrast. In this way, we cast L1 learning as *perceptual*, as in Boersma and Escudero (2008). This special status for L1 learning is warranted by results in the infant learning literature, which strongly suggest that infants learn language-specific perceptual warping before a lexicon is in place (Werker and Tees, 1984; Polka and Werker, 1994; Maye et al., 2002). An example input-output pair would be [F1 = 3.4 Bark] ~ /i/. To test whether training resulted in correct Dutch-like perception of /i/, /i/, and /ε/, we used a holdout method where the production tokens described in Section Acoustic Input Data for the Simulated Learners were first split into a training (90%) and testing (10%) subset. A total of 40,000 [acoustic] input tokens was then randomly sampled from these training sets for each learner, with the grammar updating the ranking in case of an error as described in Section Sequential vs. Interactive Processing. Following Jarosz (2013), the ranking was *resampled* (i.e., evaluation noise was applied a second time) after an error, so that the connection strengths used for parsing may differ slightly from those used for the initial evaluation.

To simulate immersion in the L2 environment, the simulated learners were next trained on labeled pairs of binned input F1 values plus invariant carrier words (Section Acoustic Input Data for the Simulated Learners), and output <lexical> forms representing a meaning congruent with the chosen carrier word and vowel token. An example input-output pair would be [tʃVka], F1(V) = 3.8 Bark] ~ <girl>. Learners were given no information about the intermediate /phonetic/ and |phonemic| categories; remapping these representations takes place only on the basis of the target <lexical> form through the parsing strategy described in Section Meaning-driven Learning, and learners began with a system optimally suited to perceiving the L1 training data.

In all other respects, L2 training resembles L1 training: again the input data were split into a training (90%) and testing (10%) subset, and a total of 40,000 training tokens (generated from the training data) was given to learners, who again employed resampling to determine which ranking values to update in case of an error. The (informal) pseudocode below summarizes the learning algorithm performed on the L1 and L2 training datasets.

for each pair (*input*_T_ ~ *output*_T_) in training set

add *evaluation noise* to ranking values of all connections
evaluate optimal path (input_T_…output_O_) from input_T_
if output_T_ ≠ output_O_
          add *evaluation noise* to all connection strengths in grammar
          evaluate target parse between input_T_ and output_T_
          decrease ranking value for each connection in optimal path by *plasticity*
          increase ranking value for each connection in target parse by *plasticity*
          decrease plasticity by decay rate

## Modeling results

Results were obtained by evaluating tokens from the test sets at various stages of L1 and L2 training. No learning took place on these test tokens. Since there are some elements of randomness in the model and training (specifically in the division of the input data into training and test sets, and the noise employed in evaluation), we ran 50 simulations for both the sequential and interactive versions of the grammar, representing 50 simulated sequential-type and 50 simulated interactive-type learners. The results reported here are averaged over these 50 simulated learners per grammar type.

### L1 learning

**Figure 6:**
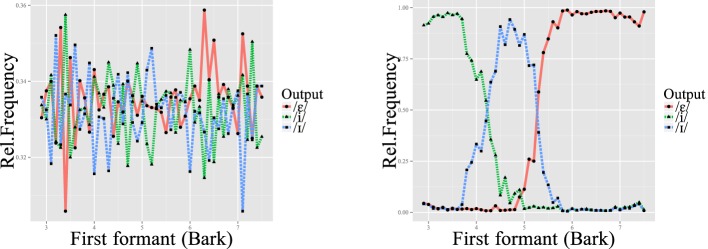
**Classification of inputs after 0 (left) and 40,000 (right) learning iterations on the L1 input data**.

**Figure 7:**
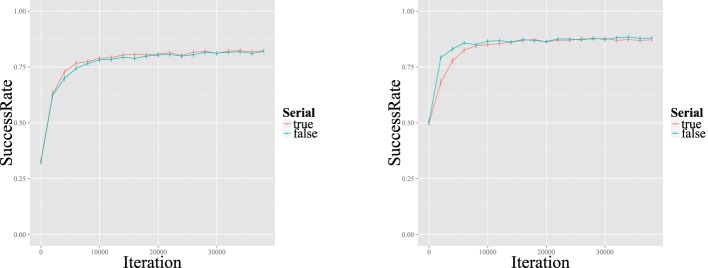
**Lexical recognition rates over time for Dutch L1 (left) and Spanish L2 (right) training**. Bars indicate 95% confidence intervals. The “true” and “false” lines stand for sequential and interactive learners, respectively.

As stated above, L2LP assumes the initial state of an L2 grammar to be a copy of the L1 grammar. We simulated this initial state by first training each grammar on the discretized acoustic values coupled to the phonetic categories mentioned above. Since the L1 training concerns only the mapping from [acoustic] inputs to the /phonetic/ level, without involving the lexicon, there is no difference in behavior between the sequential and interactive learners. Figure 6 shows how these [acoustic]-/phonetic/ mappings develop over the course of training. At the end of training, the categorization curves matched those of the input distribution of Figure 5. Since the distributions of the three vowels on the F1 continuum show some overlap, learners reached a ceiling of about 80% correct recognition of the test set (Figure 7, left). This means that without lexical or semantic context, it is not always possible to distinguish these vowels from one another.

### L2 learning

Both sequential and interactive learners were able to improve their classification of the Spanish minimal <lexical> pairs, arriving at a stable recognition rate of around 85% over time. As in L1 learning, this is probably the peak possible success rate given the fact that the distributions of Spanish /i/ and /e/ overlap, as shown in the original vowel production study (see Chládková et al., 2011 and Figure 5). Although sequential learners needed a slightly larger number of input data to attain this peak rate, both types ultimately reach this ceiling (with overlapping confidence intervals) after about 8000 iterations, as shown in Figure 7 (right). This slower attainment may be a consequence of the more L1-like representations maintained by sequential learners, as will be discussed below.

The success of this new implementation of the model in learning to recognize the L2 confirms our hypothesis that the original L2LP's predictions as implemented by Weiand (2007) failed because of the phonologically inspired “faithfulness” connections. The current revision, which implements phonetic-phonemic mappings through a more general concept of “connection strength,” is more successful in modeling empirical L2 learning results. The revised L2LP furthermore shows that the *meaning-driven* learning of lexical items proposed by Escudero (2005) can account for improved understanding of the L2 through exposure to the language.

**Figure 8:**
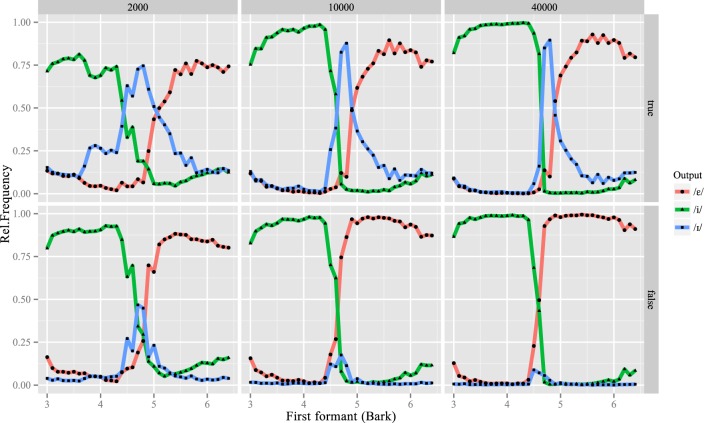
**Sequential (above) and interactive learners' /phonetic/ categorization of inputs after 2000 (left), 10,000 (middle) and 40,000 (right) learning iterations**.

Furthermore, the L2LP model makes specific predictions on learners' phonological categorization of speech sounds over the course of development. All learners shifted the boundaries between /phonetic/ categories during learning: they adapted to the two-vowel L2 system at the cost of the middle /i/ category, as shown by the /phonetic/ categorization of learners over time (Figure 8). This result of the simulations closely resembles the empirical findings of Escudero and Boersma (2002), as well as the modeling results of Boersma and Escudero (2008), which assumed learners access category labels. The revised model however shows that acquiring L2-like representations can also be modeled as *meaning-driven*, without assuming that a learner has explicit knowledge of the L2 phonological categories, an assumption that was at the core of Boersma and Escudero's (2008) model.

Without phonetic or phonemic labels in the L2 input data, learners are faced with several options on how to adapt their old perceptual systems to the L2. Interestingly, Figure 8 also shows that the sequential and interactive versions of the model do not predict the same extent of perceptual remapping in the L2. For interactive learners, the former Dutch /i/ category eventually falls into complete disuse, so that these L2 Spanish learners are effectively native-like in their *perception* of front vowels, employing only two categories. The sequential model predicts that perception of /i/ diminishes, but is retained for certain inputs. This difference in /i/ responses after learning, corresponding to the area under the /i/ curve after 40,000 training tokens, is significant between the two groups of learners^4^.

This difference between the two groups is restricted to a small range of inputs: the phonemic categorizations of the two groups are significantly different for [acoustic] inputs whose F1 lies between 4.5 and 5.1 Bark.^5^ This range corresponds to Dutch /i/ and includes the boundary between Spanish /i/ and /e/. The sequential model thus predicts that L2 perception remains filtered by the L1 for these intermediate vowels, with more open vowels usually classified as /e/ but occasionally as /i/. Despite this maintenance of a three-vowel system in their internal L2 representations, sequential learners attain the same recognition rate of Spanish lexical items (Figure 7). These learners appear to consider /i/ an “allophone” of /e/ in Spanish, and store both phones as possible realizations for words containing phonemic |e|. We discuss the implications of these predictions below.

## Discussion

Experience in one's native language largely shapes the perceptual and lexical acquisition of a second language. We provide a computational, network-like model of L2 perception and lexicalization. The revised L2LP retains (psycho) linguistic concepts on representations and evaluation of input data, but removes a number of assumptions from theoretical phonology about the way units on these levels of representation are connected. Discarding these assumptions has increased the explanatory power of the model, suggesting that a strictly symbolic view of the phonetics-phonology interface is not consistent with what we know about L2 learning. Another novel aspect was that we trained our simulated learners on data taken directly from vowel production studies, rather than artificial distributions.

Our first aim was to explore the viability of a *meaning-driven* learning paradigm, in which learners have access to the intended meanings but not to the phonological specifications of the L2 input. Simulated learners showed progress toward native-like perception and recognition of front vowels, progressively adapting to the L2 in a way similar to real-life L2 learners (Escudero and Boersma, 2002). This mirrors the results of an earlier modeling study (Boersma and Escudero, 2008) but obviates the assumption that overt phonological structure is present in the learning input.

Secondly, the revised model allows us to differentiate between a sequential and an interactive perspective on phonetic (pre-lexical) perception and lexical recognition. While both versions of the model gravitate toward correct recognition of the L2, they make different predictions on the phonetic representations ultimately employed by learners. Specifically, sequential learners are predicted to retain an L1 phonetic category for certain “boundary” stimuli whereas interactive learners ultimately fully adapt their vowel system to the L2. Anecdotal evidence suggests that adult L2 learners only very rarely reach native-like ability, which at first glance seems more in line with the results of our sequential learners (but see Bongaerts, 1999). However, experimental evidence is needed in order to untangle the influence of L1 on the *perception* of L2 learners. Previous research (e.g., Escudero and Boersma, 2002; Mayr and Escudero, 2010; Escudero et al., 2012) have studied L2 categorization behavior by activating listeners' L2 *language mode* (Grosjean, 2000). We conjecture that categorical perception effects (discrimination peaks) in the region of the old L1 phonetic categories (e.g., the subsumed Dutch /i/) when perceiving the L2 may provide clues for the accuracy of either the sequential or the interactive model. These effects may be measured with discrimination and identification experiments, presenting the relevant tokens to advanced Dutch learners of Spanish in their Spanish language mode^6^. Experiments can include more sensitive measures such as reaction times or event-related potentials to examine whether retaining the extra L1 vowel category negatively affects L2 perception. Indeed, previous studies have shown that the availability of extra phonetic categories affects native and non-native vowel perception (Benders et al., 2012; Elvin et al., 2014). Our results thus offer testable hypotheses that may in turn contribute to the general debate of sequential vs. interactive language processing (Norris et al., 2000; McClelland et al., 2006).

We conclude that L2LP offers a workable and fruitful model of the processes underlying acquisition of non-native sound systems. Compared to alternative models of L2 acquisition, the simulation paradigm illustrated in this study allows L2LP to make very specific predictions on how L1 experience and L2 input shape the outcome of learning. These numerical predictions can be compared to empirical findings and in turn inform new hypotheses. Future work is to investigate whether L2LP's success extends beyond the subset scenario described above—for instance, the reverse scenario (which would be an instance of the L2LP new scenario) of going from a two-way to a three-way contrast, and would therefore require the creation of a new L2 category rather than the discontinued use of an old L1 category.

### Conflict of interest statement

The authors declare that the research was conducted in the absence of any commercial or financial relationships that could be construed as a potential conflict of interest.

## Footnotes

^1^Escudero and Boersma (2002) did not collect goodness of fit ratings together with L1 categorization, which is crucial for establishing the perceptual assimilation types proposed within PAM, which are the start point for the model's discrimination difficulty predictions. However, following Bundgaard-Nielsen et al. (2011), one can conclude that Spanish /i/-/e/ are categorized or assimilated to the Dutch contrast /i/-/i/, given that an L2 vowel is defined as *categorized* if it was identified as an L1 vowel in more than 50% of presentations.

^2^Escudero (2005)'s original proposal considered learning *message-driven*, but as the learning data for the model described in sections §2 and §3 do not strictly contain *messages*, we use the term *meaning-driven* here.

^3^Traditionally in BiPhon and L2LP these four levels are known as *Auditory, Surface, Underlying*, and *Lexical* Form, respectively. Here we replace these phonological concepts with terms more familiar to psychologists and psycholinguists.

^4^Welch's *t* -test, two-sided, *t* = 30.5903, df = 69.32, *p* < 2.2e−16 (smallest floating point number representable in R).

^5^Per input F1 value, relative frequency of response for each vowel was summed per learner type (sequential vs. interactive); Pearson's chi-squared (response × type) was significant (N1 = N2 = 50, df = 2, χ ^2^ = 7.06, Bonferroni-corrected *p* = 0.00047).

^6^A reviewer suggested the possibility that either strategy occurs in real-life L2 learners, and is perhaps a locus of individual differences in L2 acquisition. The potential co-existence of the two types of grammars in the same listener or differences across listeners can also be explored with the proposed experiments.

## Appendix

### Minimal pairs used as target lexical items

**Table:**

This List is Identical to that Used in Weiand, 2007.
checa “Czech.F” chica “girl”
checo “Czech.M” chico “boy”
fecha “date” ficha “token”
gres “stoneware” gris “gray”
lega “layman” liga “league”
lema “motto” lima “file”
meca “Mecca” mica “mica”
mesa “table” misa “Mass”
memo “fool” mimo “mime”
reto “dare” rito “rite”
rezo “prayer ” rizo “curl”
veda “prohibition” vida “life”
peso “weight” piso “floor”
